# Global health in Canada: Three emerging prospects

**DOI:** 10.7189/jogh.11.03025

**Published:** 2021-01-30

**Authors:** Michelle Amri

**Affiliations:** 1 Dalla Lana School of Public Health, University of Toronto, Toronto, Ontario, Canada; 2 Takemi Program in International Health, Harvard T.H. Chan School of Public Health, Boston, Massachusetts, USA; 3 School of Public Health and Social Policy, University of Victoria, Victoria, British Columbia, Canada

“Global health” means different things to different people. While there is an academic dialogue around what the field entails, developing from its roots of hygiene & tropical medicine and international health, there is still room for enhanced understanding and refined scope [1]. This is particularly the case with the COVID-19 pandemic, which has brought public and global health considerations into almost every decision.

The recent 26^th^ Canadian Conference on Global Health (CCGH), convened virtually from October 19 to 22, 2020 by the Canadian Society for International Health, was a key venue for further examining the different facets of global health. Featuring renowned speakers from numerous sectors, such as Drs. Jane Goodall, Anthony Fauci, and Tedros Adhanom Ghebreyesus, discussions ranged greatly from the humanitarian response to COVID-19 to changing politics. The conference allowed for a reimagined understanding of the field and paved the way to a broader understanding that included three emerging prospects: emphasizing the importance of policy and governance, encouraging engagement of participants from the global south, and elevating the voice of women.

In 2006, Dr Ilona Kickbusch observed in the Canadian Journal of Public Health that “[t]*he present global health crisis is not primarily one of disease, but of governance: its key characteristic is a weakening of public policy and interstate mechanisms as a consequence of global restructuring*” [2]. Nearly fifteen years later, this remains true. Reflecting on COVID-19, while discussions have largely centred on disease dynamics, there is growing attention on governance and public policy. Dr Kickbusch eloquently reminded us during her CCGH session, *Moving to the Future of Health Promotion*, that “health is always about political choices and health is always about power. These power shifts have to be addressed by health promotion”. She reminded us that in health promotion, we are not focused on disease, and look to health and well-being instead. As a discipline and approach, health promotion encourages the living of a good life, and health as a resource for everyday life. Further, Dr Kickbusch draws attention to the fact that our thinking in the global north is only now catching up with the global south where well-being has been considered for hundreds of years.

The 26^th^ CCGH delivered virtually afforded opportunities for over a thousand participants from around the world to attend; drawing participation from: Afghanistan, Algeria, Argentina, Bangladesh, Belgium, Benin, Botswana, Brazil, Burkina Faso, Cameroon, Canada, Colombia, Costa Rica, Democratic Republic of the Congo, Denmark, Dominican Republic, France, Gambia, Georgia, Germany, Ghana, Guinea, Ethiopia, Egypt, Haiti, Honduras, Hungary, India, Indonesia, Iraq, Kazakhstan, Kenya, Libya, Madagascar, Malaysia, Malawi, Mali, Mexico, Morocco, Mozambique, Myanmar, Nepal, Netherlands, Nigeria, Pakistan, People’s Republic of China, Peru, Philippines, Portugal, Rwanda, Saint Lucia, Senegal, Spain, Somalia, South Africa, South Sudan, Sri Lanka, Switzerland, Turkey, United Republic of Tanzania, Uganda, United Kingdom of Great Britain and Northern Ireland, United States of America, Viet Nam, Zambia, and Zimbabwe. With more diverse voices and perspectives from around the world, global health practice can be improved. This is particularly the case with different cultures which may have different understandings and perspectives, which draw upon different traditions of social justice [3]. For example, in considering equity, it has been suggested that the United States of America focuses on racial or ethnic disparities whereas the United Kingdom focuses on socioeconomic status [4]. Given the shared interest in global health, which largely focuses on the global south, it is crucial to have global health representation and engagement in such discussions. Despite high-income countries only accounting for 17% of the global population, a study of 200 global health organizations found that 83% of global health leaders are from high-income countries [5].

**Figure:**
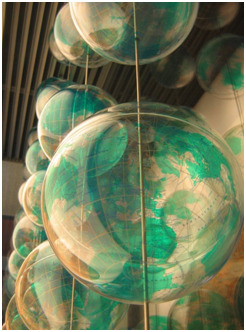
Photo: “Globes” by Tuppus; licensed under CC BY 2.0.

Similarly, with 49.6% of the global population identifying as female in 2017 [6], the female voice also needs to be heard and listened to. This is particularly important with the large percentage of young females engaged in global health, but with a low percentage successfully moving to leadership positions [7]. In the same study of 200 global organizations, it was determined that only 5% of leaders were women from low- and middle-income countries [5], which demonstrates the dire need to elevate these voices. This was also found to be the case more recently, as it was determined by *The BMJ Gender Diversity Group* that women were underrepresented in decision-making around COVID-19 [8]. The 26^th^ CCGH unveiled the 2020 Canadian Women in Global Health list, “showcasing the incredible achievements of Canadian women leaders in global health, fostering new and exciting collaborations, and advocating for gender equity in the health workforce and leadership more broadly” [9]. In speaking about the list at CCGH, Eva Slawecki, Executive Director of the Canadian Society for International Health, expressed that “there is no excuse to say there is no women expert”. This list allows for enhanced gender representation and engagement in global health, by providing an easily searchable database of women experts which can be drawn upon. This is crucial because lack of gender representation translates to a lack of action to address causes of gender-based health inequities [5]. For instance, when examining the COVID-19 pandemic, countries with female leaders fared much better across various indicators, including having six times less deaths [10]. The list is a step towards paving the way for women leaders and reimagined approaches to global health.

Overall, I must commend the CCGH and its organizers for outlining three emerging prospects for which global health can be reconsidered. With enhanced attention paid to global health, we can reform our approaches, through emphasizing the importance of policy and governance, encouraging engagement of participants from the global south, and elevating the voice of women, to ultimately enhance public health to protect populations and improve public health.
